# How to Surgically Remove the Permanent Mesh Ring after the Onstep Procedure for Alleviation of Chronic Pain following Inguinal Hernia Repair

**DOI:** 10.1155/2016/5209095

**Published:** 2016-05-19

**Authors:** Stina Öberg, Kristoffer Andresen, Jacob Rosenberg

**Affiliations:** Center for Perioperative Optimization, Department of Surgery, Herlev Hospital, University of Copenhagen, Herlev Ringvej 75, 2730 Herlev, Denmark

## Abstract

A promising open inguinal hernia operation called Onstep was developed in 2005. The technique is without sutures to the surrounding tissue, causing minimal tension. A specific mesh is used with a memory recoil ring in the border, which may cause pain superficial to the lateral part of the mesh for slender patients. The aim of this study was to illustrate an easy procedure that alleviates/removes the pain. A male patient had persistent pain six months after the Onstep operation and therefore had a ring removal operation. The procedure is presented as a video and a protocol. At the eleven-month follow-up, the patient was free of pain, without a recurrence. It is advised to wait some months after the initial hernia repair before removing the ring, since the mesh needs time to become well integrated into the surrounding tissue. The operation is safe and easy to perform, which is demonstrated in a video.

## 1. Introduction

Inguinal hernia surgery is a common procedure [1] and it is estimated that surgeons worldwide perform more than 20 million inguinal hernia repairs annually [2]. The most commonly used surgical techniques are the laparoscopic techniques and the open Lichtenstein procedure, but they have problems such as recurrences and chronic pain [3]. In 2005, two surgeons developed a new open inguinal hernia operation called Onstep [4]. The mesh used for this technique is made of polypropylene, with a memory recoil ring in the border of the mesh (PolySoft Mesh-Bard, Davol Inc., Warwick, RI). Studies have shown promising results for the Onstep operation, with both a low chronic pain rate and few recurrences [4, 5]. The mesh is laterally secured around the spermatic cord, and medially it lies in the preperitoneal area, but no sutures are used to secure the mesh to the surrounding tissue. The ring prevents the mesh from folding and helps keeping it in place. Lateral to the spermatic cord, the only structures separating the mesh from the skin are subcutaneous fat and the fascia of the external oblique. In our department, a few slender patients have had pain or discomfort due to pressure of the ring in the mesh against the skin [5]. The inventors of the Onstep technique also observed this complication among a few patients (personal communication), but the pain disappeared after a simple procedure where the memory ring was removed [4].

Because of the promising results of the Onstep operation, it is expected that more surgeons will start to use this new technique for inguinal hernia repairs. Therefore the numbers of patients with complaints from the ring may rise and it is important that surgeons know how to remove the ring in the mesh.

The aim of this study was to provide a surgical video of the procedure, which shows surgeons how to quickly and easily alleviate or remove the pain for affected patients. The video demonstrates how to remove the ring from the mesh, without increasing the risk of an inguinal hernia recurrence [4].

## 2. Case Presentation

The patient had his primary Onstep operation six months prior to the ring removal operation, and his Body Mass Index (BMI) was 19.6 kg/m^2^. The initial Onstep operation was performed to treat a primary inguinal hernia. After the operation, the ring in the mesh gave rise to a prominence laterally in the groin area, which caused daily pain that required an elective ring removal operation under general anesthesia. The ring removal procedure can be performed under local anesthesia as well [4], but since there may be pressure on the spermatic cord when removing the ring in the mesh, with resulting pain, general anesthesia is preferred. After the ring removal procedure, we conducted two telephone interviews at one and four months, respectively. The last follow-up was by mail, eleven months after the operation.

### 2.1. Surgical Procedure

The ring removal procedure is visualized and vocally explained in the video. The procedure is performed through a small incision on top of the prominence and dissection is done to the space where the mesh lies, between the internal and external oblique muscles. The lateral part of the mesh is then visualized. The next step is to identify the ring in the mesh. The ring is placed in a drawstring in the border of the mesh and is divided laterally, so that the two ends make a separate fold inside the mesh. The surgeon should cut in the mesh laterally, until one or both ends of the ring are visualized and grasp one end of the ring with a clamp. The ring can thereafter be pulled out without removing the rest of the mesh. It may be necessary to use some force to remove the ring since the mesh is well integrated in the surrounding tissue. Finally, the only steps remaining are to suture the fascia of the external oblique muscle and then the skin.

The video is available as a supplemental file; see Video 1 in Supplementary Material available online at http://dx.doi.org/10.1155/2016/5209095.

## 3. Results

The prominence disappeared after removal of the ring, and the patient could return to work the same day. Preoperatively, the patient had daily pain, especially when wearing trousers. At one-month follow-up, the only pain left was when coughing heavily (the patient suffers from chronic lung disease) and the patient had no sign of a hernia recurrence. At the four- and eleven-month follow-up, the pain when coughing heavily was gone. There was still no sign of a recurrence. Overall, the patient was very satisfied with the operation.

## 4. Discussion

The patient in this study had a surgical removal of the ring in a permanent hernia mesh, six months after the Onstep operation. The video demonstrates the technique of how to remove the ring, with an explaining voice-over. The preoperative pain from the protruding ring was gone at the last follow-up, without development of an inguinal hernia recurrence.

The Onstep operation has existed for more than 10 years and is now introduced to several European countries and very soon to the rest of the world. Advantages of this open operation are that it is easy to learn and the duration of the procedure is approximately 15 minutes. Studies have also shown that both chronic pain and recurrence rates are low, the latter ranging from 0.6% [4] to 3.8% [5]. Today there are only two studies about the Onstep operation reported in English language [4, 5], but since the results are promising, we expect an increased use of the technique in the years to come, and there are several ongoing clinical trials. It has come to our knowledge that especially slender patients may develop pain from the ring in the mesh, where it laterally lies very superficially if the patient has a small amount of subcutaneous fat. When the lateral part of the ring, and/or trousers, creates pressure on the skin, patients may develop pain or discomfort. The patient in this study had chronic pain, since the pain persisted 6 months after the initial Onstep operation [6]. However, the attached video in this study teaches the technique of how to decrease or remove the pain, and therefore chronic pain arising from a protruding ring can be dealt with. Thus, it is important for surgeons to be aware of this complication following the Onstep operation and to learn the procedure, since the cause of the pain can be surgically removed [4].

Before performing the procedure of ring removal, the surrounding connecting tissue needs time to become well integrated into the mesh. This is important to ensure that the mesh stays in place when the ring is pulled out. One study showed that, after 10 weeks, tissue integration was very good for polypropylene [7]. We normally recommend waiting six months before removal of the ring is indicated, both to ensure that the mesh stays in place and to make sure that the pain does not diminish by itself, as was seen for some patients in the Portuguese study [4]. However, timing of the removal of the ring needs to be based on an individualized assessment.

The strength of this study was that the video illustrates the ring removal procedure well, and the technique is also explained by voice-over. The limitation of the study was that the video was obtained by the authors and not by a professional film crew. This is a case-report with the purpose of illustrating how to perform the ring removal procedure. The pioneers of the Onstep technique have removed the ring on three of the four patients with pain following Onstep, and the pain disappeared without any recurrence (one-year follow-up) [4]. The authors in this paper have a submitted case-series of the Onstep procedure, where six patients with a ring removal had alleviation or disappearance of their complaints, without a recurrence (a couple of months postoperatively).

In conclusion, the video demonstrates a safe and easy way to remove the ring in the mesh. Eleven months after the operation, the patient was pain-free and without a recurrence of the hernia.

## Supplementary Material

Video 1: Demonstration of how to surgically remove the ring in the PolySoft Mesh without removing the rest of the mesh, six months after the initial hernia repair. Also available at: .
